# Hemodynamic effects of HPMA copolymer based doxorubicin conjugate: A randomized controlled and comparative spectral study in conscious rats

**DOI:** 10.1080/17435390.2017.1285071

**Published:** 2017-02-09

**Authors:** Hoay Yan Cheah, Olivera Šarenac, Juan J. Arroyo, Marko Vasić, Maja Lozić, Sofija Glumac, See Ziau Hoe, Charles Colin Thomas Hindmarch, David Murphy, Lik Voon Kiew, Hong Boon Lee, María J. Vicent, Lip Yong Chung, Nina Japundžić-Žigon

**Affiliations:** a Department of Pharmacology, Faculty of Medicine, University of Malaya, Kuala Lumpur, Malaysia;; b Institute of Pharmacology, Clinical Pharmacology and Toxicology, Faculty of Medicine, University of Belgrade, Republic of Serbia;; c Polymer Therapeutics Lab, Centro de Investigación Príncipe Felipe, Valencia, Spain;; d Department of Physiology, Faculty of Medicine, University of Malaya, Kuala Lumpur, Malaysia;; e Department of Biomedical and Molecular Sciences, School of Medicine, Queen's, University, Kingston, ON, Canada, UK;; f Molecular Neuroendocrinology Research Group, The Henry Wellcome Laboratories for Integrative Neuroscience and Endocrinology, University of Bristol, Bristol, England, UK;; g Department of Pharmacy, Faculty of Medicine, University of Malaya, Kuala Lumpur, Malaysia

**Keywords:** Nanomaterials, polymer carriers, telemetry, echocardiography, cardiotoxicity

## Abstract

Conjugation of Doxorubicin (DOX) to *N*-(2-hydroxypropyl) methylacrylamide copolymer (HPMA) has significantly reduced the DOX-associated cardiotoxicity. However, the reports on the impact of HPMA–DOX conjugates on the cardiovascular system such as blood pressure (BP) and heart rate (HR) were in restrained animals using tail cuff and/or other methods that lacked the resolution and sensitivity. Herein, we employed radiotelemetric-spectral-echocardiography approach to further understand the *in vivo* cardiovascular hemodynamics and variability post administration of free DOX and HPMA–DOX. Rats implanted with radio-telemetry device were administered intravenously with DOX (5 mg/kg), HPMA–DOX (5 mg DOX equivalent/kg) and HPMA copolymer and subjected to continuous cardiovascular monitoring and echocardiography for 140 days. We found that DOX-treated rats had ruffled fur, reduced body weight (BW) and a low survival rate. Although BP and HR were normal, spectral analysis indicated that their BP and HR variabilities were reduced. All rats exhibited typical signs of cardiotoxicity at histopathology. In contrast, HPMA–DOX rats gained weight over time and survived. Although BP, HR and related variabilities were unaffected, the left ventricular end diastolic volume (EDV) of these rats, as well as of the HPMA copolymer-treated rats, was found increased at the end of observation period. Additionally, HPMA copolymer caused microscopic injury of the heart tissue. All of these suggest the necessity of caution when employing HPMA as carrier for prolonged drug delivery. The current study also indicates the potential of radiotelemetric-spectral-echocardiography approach for improved preclinical cardiovascular risk assessment of polymer–drug conjugate and other nano-sized-drug constructs.

## Introduction

Doxorubicin (DOX) is an effective anti-neoplastic drug used in the treatment of solid malignant tumors, leukemia, lymphomas and breast cancer (Katzung et al., 2004). However, its use is hampered by narrow therapeutic index, organ toxicity and the development of cardiomyopathy that may occur several years after termination of the treatment, preventing prolonged courses of chemotherapy. The development of cardiomyopathy is unpredictable; it is resistant to treatment and is associated to high mortality rate (Chatterjee et al., 2010; Octavia et al., 2012).

Many strategies have been developed to overcome the problem of DOX-induced cardiotoxicity. Clinical biomarkers monitoring (Cardinale et al., 2004; Dolci et al., 2008), strain echocardiography (Curigliano et al., 2012; Sawaya et al., 2011), heart rate (HR) variability assessment (Lončar-Turukalo et al., 2015), and radionuclide ventriculography (Sipola et al., 2012) have been used for the early diagnosis of subclinical forms of cardiotoxicity, in order to discontinue the treatment before irreversible damage occurs. Also, preventive treatments were attempted through the use of dexrazoxane that interferes with iron-mediated free radical generation and carvedilol, a vasodilating β blocker with antioxidant activity, but none seems to be effective enough (Mitry & Edwards, 2016).

In the last few decades nanodrug carrier systems have been developed for targeted delivery of anti-cancer drugs and to reduce systemic toxicity (Yokoyama, 2014). Nano carriers are biocompatible macromolecules, with low immunogenicity, that evade renal filtration and achieve long blood circulation time. *N*-(2-hydroxypropyl) methacrylamide (HPMA) copolymer is one of such system that delivers its drug payload passively via the enhanced permeability and retention (EPR) effect (Kedar et al., 2010; Kopeček et al., 2000). To reduce the systemic toxicity and enhance the tumor delivery of DOX, HPMA copolymer has been combined with DOX via a Gly–Phe–Leu–Gly peptide spacer to form HPMA copolymer–DOX conjugates. In a few preclinical (Hopewell et al., 2001; Yeung et al., 1991) and clinical studies (Seymour et al., 2009; Vasey et al., 1999) it has been suggested that DOX’s *in vivo* cardiotoxicity profile has been improved by conjugation to HPMA copolymer. However, no data are available about cardiovascular hemodynamic profile of HPMA copolymer bound to DOX and HPMA copolymer alone in respect to the free drug. Since large molecules such as HPMA copolymer may affect cardiovascular hemodynamic which, in turn, may trigger autonomic nervous system response we sought to investigate effects of HPMA bound DOX in respect to free DOX on cardiovascular hemodynamic and short-term variability in freely moving rats. We used radio-telemetry and echocardiography approach for hemodynamic parameter assessment and spectral analysis of blood pressure (BP) and HR to evaluate autonomic cardiovascular control.

## Methods

### Materials

HPMA copolymer Gly–Phe–Leu–Gly–ONp (5 mol %; *Mw*∼20,000–25,000 g/mol and *Mw*/*Mn* = 1.3–1.5) was obtained from Polymer Laboratories Ltd, Shropshire, UK. DOX (hydrochloride) was purchased from Xingcheng Chempharm CO., Ltd., Zhejiang, China. All the chemicals were purchased from Sigma-Aldrich Chemie, Germany. Ketamine, xylazine, acepromazine and tetracaine (T61^®^ injection solution) injections were purchased from MarloFarma (Belgrade, Republic of Serbia). Carprofen and gentamicin injections were purchased from Hemofarm (Vršac, Republic of Serbia).

### Synthesis of HPMA copolymer–DOX conjugate

Adapted from previously reported (Vicent et al., 2005). In summary, one equivalent of the precursor (HPMA copolymer–GFLG–ONp) and DOX·HCl were dissolved in the minimum amount of dry DMF, under N_2_ stream and stirring in a round bottomed flask. Triethylamine (one equivalent) was added drop wise to the copolymer solution in order to neutralize the hydrochloric acid to give DOX as a free amine. The reaction was allowed to proceed at room temperature overnight under N_2_ atmosphere and then quenched by adding 1-amino-2-propanol. The reaction was monitored by measuring aliquots at different time points by UV (release of ONp at 400 nm) and by TLC (mobile phase: acetic acid/butanol/water (0.5:6.5:3.0); Rf, DOX =0.55; Rf, polymer =0) as described by Vicent et al. (2005).

DMF was evaporated under reduced pressure and the resultant solid was dissolved in HPLC-grade methanol (20 mg/ml). The polymer–drug conjugate was filtered and purified by size exclusion chromatography using consecutive columns of Sephadex LH-20 (3 × 50 cm) with HPLC-grade methanol as the eluent. The purified compound was then dissolved in a minimal amount of water and freeze-dried. The overall yield based on polymer weight was around 75%.

### Characterization of HPMA copolymer–DOX

#### Determination of total DOX loading by UV

Briefly, samples for UV determination were prepared in triplicate by dissolving the dry solid conjugate in HPLC-grade methanol (1 mg/mL) and dilutions of 0.1, 0.25 and 0.5 mg/mL were measured at 480 nm. The resulting absorbance was compared with a calibration curve of DOX (0.005–0.065 mg/mL) in HPLC-grade methanol determined under the same experimental conditions.

#### Determination of total DOX loading (TDL) by HPLC

RP-HPLC analysis of the final product was analyzed following the protocol previously described (Vicent et al., 2005). Briefly, samples were dissolved in buffered milliQ water (Merck Millipore, Darmstadt, Germany) (1 mg/ml) and DOX aglycone was obtained by acidic treatment to cleave the acetal bond in DOX. A liquid–liquid extraction procedure was then carried out, the supernatant phase was discarded and the organic phase was dried. For HPLC sample preparation, the dry residue was re-dissolved in HPLC-grade methanol and fluorescence was measured by an in-line detector (*λ*_ex _=_ _480 nm, *λ*_em _=_ _560 nm) and compared with a calibration curve of DOX in the range between 0.005 and 0.065 mg/ml previously determined in the same experimental conditions.

#### Determination of free DOX content by HPLC

The same aqueous solutions prepared for TDL determination was used. Briefly, fresh solutions of the conjugate were prepared in buffered milliQ water (1 mg/mL) and the same liquid–liquid extraction described previously as well as sample treatment was carried out. The obtained fluorescence by HPLC was compared with a calibration curve of DOX previously determined in the same experimental conditions (Vicent et al., 2005).

#### Size by gel permeation chromatography (GPC)

HPMA copolymer was analyzed using DMF phase GPC. Polysaccharide standards (pullulan) (*Mw* from 11,800 to 210,000 g/mol) were used to generate a calibration curve. All samples were prepared in DMF containing 1% LiBr in a final concentration of 8 mg/mL and 100 μL aliquots were injected using a flow ratio of 0.8 mL/min. Both RI and viscosity detectors were used.

### Experimental animals and study design

All experimental procedures conformed to European Communities Council Directive of November 24, 1986 (86/609/EEC) and were approved by the University of Belgrade Ethics review board.

Experiments were performed in 11 weeks old out-bred male Wistar rats (300 g ± 10) bred in local animal facility of the Faculty of Medicine University of Belgrade. Rats were randomly allocated into 4 treatment groups. In HPMA copolymer group rats (*n* = 6) received HPMA copolymer (65.8 mg HPMA/kg, equivalent to the amount of HPMA copolymer present in a dose of 5 mg DOX equivalent/kg of HPMA–DOX, i.v.). In SALINE group, rats (*n* = 6) were treated with 0.9% NaCl (0.5 mL; i.v.). In HPMA copolymer–DOX conjugate group, rats (*n* = 6) received HPMA copolymer–DOX conjugate (5 mg DOX equivalent/kg, equivalent to 65.8 mg HPMA–DOX/kg; i.v.) and in DOX group (*n* = 11 rats), free doxorubicin (5 mg DOX/kg, i.v.). During experimentation rats were maintained under standard laboratory conditions (12:12 light–dark cycle, ambient temperature 22 °C ± 1 and relative humidity 65% ± 5) with free access to rodent chow and tap water. After implantation of radiotelemetric probe for continuous cardiovascular recording, rats were observed for 140 days. Rat body weight (BW), BP, HR, BP short-term variability (BPV), HR short-term variability (HRV), left ventricular ejection fraction (EF_LV_) and left ventricular end-diastolic volume (EDV) were monitored weekly. At the end of the follow-up period, rats were sacrificed and the hearts were harvested for histopathology.

### Assessment of general toxicity

General toxicity was assessed by observation of rat appearance, behavior, BW changes and survival rate, as well as necropsy and histopathology. Single dose of 5 mg/kg i.v. of DOX was taken from literature (Duncan et al., 1998; Yeung et al., 1991) and verified in pilot experiments.

### Surgical implantation of radiotelemetric probes

Rats were anesthetized by combined ketamine (100 mg/kg, i.p.) and xylazine (10 mg/kg, i.p.) anesthesia. They were placed on a heating pad in supine position and a small thermistor was inserted into the rectum to monitor the body temperature throughout surgery. Upon shaving and disinfecting abdominal area, a 3 cm-long ventral midline incision was made. After retracting intestines, abdominal aorta was exposed. The tip of the catheter from the radiotelemetric probe (TA11PA-C40; Data Science International (DSI), St. Paul, MN) was inserted into the aorta, fixed with 3M Vetbond^TM^ and tissue cellulose patch (DSI, St. Paul, MN). The transmitter was attached to the anterior abdominal wall and the wound was closed by two layer suturing. To prevent infection, the skin suture was sprayed by topical bacitracin, neomycin, and the each rat received gentamicin (25 mg/kg, i.m.) for three days prior to surgery and on the day of surgery. For pain relief rats received carprofen (5 mg/kg/day, s.c.) on the day of surgery and for the next two days. During recovery period rats were individually housed in a Plexiglas cage (30 cm ×30 cm ×30 cm) under controlled laboratory conditions for 10 days prior to experimentation.

### Radiotelemetric monitoring of the cardiovascular parameters

Rats housed in individual cages were positioned on top of the RPC-1 telemetry receivers (DSI, St. Paul, MN) and monitored weekly throughout the 140-days-long study. Arterial BP was digitalized at 1000 Hz using Dataquest A.R.T. 4.2 software (DSI, St. Paul, MN). Systolic (SBP) and diastolic (DBP) BP and HR were derived from the arterial pulse pressure as maximum, minimum and inverse distance between successive *dP*/*dt*_max_ of the pulse pressure wave, respectively (Figure S1). Mean blood pressure (MBP) was calculated as integral of the arterial pulse pressure waveform.

### Spectral analysis of SBP, DBP and HR short-term variability

Systolic BP (SBP) and diastolic BP (DBP) and HR signals were re-sampled at 20 Hz and subjected to nine-point Hanning window filter and linear trend removal. Spectra were obtained using a fast Fourier transform (FFT) algorithm on 15 overlapping 2048-point time series corresponding to a 410 s (∼7 min) registration period of SBP and DBP and HR. The power spectrum of BP (mmHg^2^) and HR (bpm^2^) for 30 FFT segments was calculated for the whole spectrum (0.0195–3 Hz) and in the following three frequency ranges: very low frequency (VLF, 0.01–0.2 Hz), low frequency (LF, 0.2–0.8 Hz) and high frequency (HF, 0.8–3 Hz). The LF oscillation in SBP and DBP spectra (LF SBP and LF DBP) and LF/HF HR are recognized clinical markers for sympathetic modulation of vascular tone and sympatho-vagal balance to the heart, respectively (Japundzic-Zigon, 1998).

### Echocardiography

Conscious rats tranquilized with acepromazine (0.5 mg/kg, i.m.) were examined by echocardiography using a commercial echocardiograph ALOKA ProSound 2 with 13 MHz linear probe (Hitachi Medical Systems Europe, Zürich, CH, Switzerland) before treatment (day 0) and every week after treatment by different compounds. All parameters were taken from the right parasternal short axis. Analysis was carried out in cardiac volume and function analytical system in M mode. Measured parameters were: interventricular septum in diastole (IVSd), left ventricular end-diastolic internal diameter at Q wave (LVIDd), posterior wall thickness at diastole (PWd), interventricular septum in systole (IVSs), left ventricular end-systolic internal diameter at T wave (LVIDs) and posterior wall thickness at systole (PWs). Other parameters were calculated as follows: end diastolic volume (EDV) (mL) = 1.047·(LVIDd)^3^ and left ventricular ejection fraction EF_LV_(%) = (SV/EDV)·100 where SV is stroke volume calculated as difference between EDV and end systolic volume [ESV(mL) = 1.047·(LVIDs)^3^].

### Histopathology

At the end of experiments rats were euthanized with T61^TM^ injection (150 mg/kg, i.p.). The hearts were harvested to be fixed in 4% formalin for 48 h and were dissected symmetrically into 3 mm segments. The cardiac tissues were then dehydrated in graded ethanol, embedded in paraffin blocks, cut in 3-μm-thick sections and mounted onto the glass slides. After hematoxylin-eosin (HE) staining, and Masson’s Trichrome staining for fibrous tissue, the micrographs were observed under Leica DM400 M optical microscope (Leica Microsystems, Germany).

#### Immunohistochemical staining for detection of apoptosis

Caspase 3 immunostaining was preformed according to the supplier’s instructions. Five-micrometer cut sections from tissue microarray blocks were deparaffinized, rehydrated, placed in 3% H_2_O_2_ for 10 min to block endogenous peroxidase activity, and washed with tap water. Sections were then processed with 0.01 citrate buffer (pH 6.0) and treated in a microwave oven for 20 min at 600 W and placed in a bath of tap water for 20 min, then in distilled water and in TBS buffer (pH 7.6) for 5 min, and placed in diluted goat serum for 10 min. Afterwards, the tissue sections were incubated for 1 h with the anti-Caspase three mouse monoclonal primary antibody (clone CPP32, 1:50 dilution, Leica Biosystems, Newcastle, UK). Streptavidin–biotin method using DAKO’s LSAB ± kit (DAKO, Denmark) was applied, with diaminobenzidine (DAB) as the chromogen solution and Mayer’s hematoxylin for the counterstain. Cytoplasmic staining of cardiomyocytes to apoptosis was evaluated in respect to lymphocytes that served as a positive control, and pure antibody diluent incubation (without the primary antibody) as a negative control.

### Statistical analysis

Parameters are shown as means ± SEM, and differences between the means were compared by two-way ANOVA for repeated measures followed by Bonferroni *post hoc* test using the GraphPad Prism 4.0 software (Graph-Pad Software Inc., San Diego, CA). Statistical significance level was set at *p* < 0.05.

## Results

### Characterization of HPMA copolymer–DOX

The model HPMA copolymer–DOX conjugate was synthesized as previously reported (Vicent et al., 2005) and fully characterized showing a total DOX loading (TDL) of 7.6 wt% with a free DOX content of 0.8 wt% from the total loading. Molecular weight (*Mw*) was determined as 28,000 g/mol with a polydispersity index (PDI) of 1.3.

### General toxicity of different compounds

**Figure 1.**
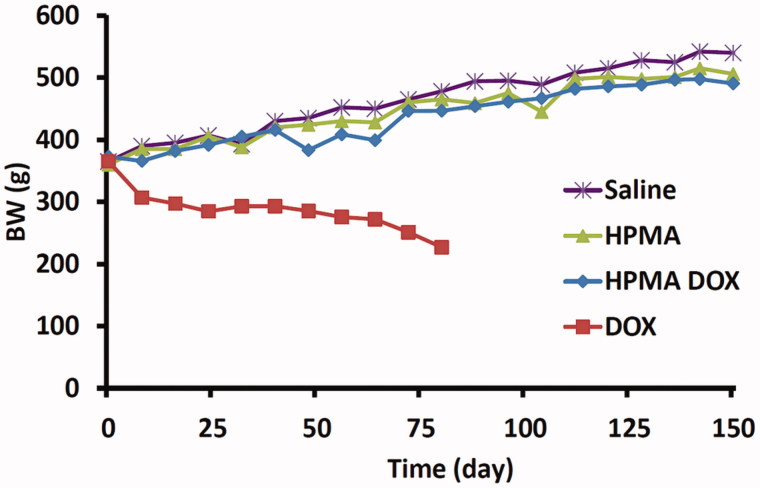
Body weight changes of Wistar rats treated with different compounds. Note that only rats treated with DOX had a decrease in body weight over time.

**Figure 2.**
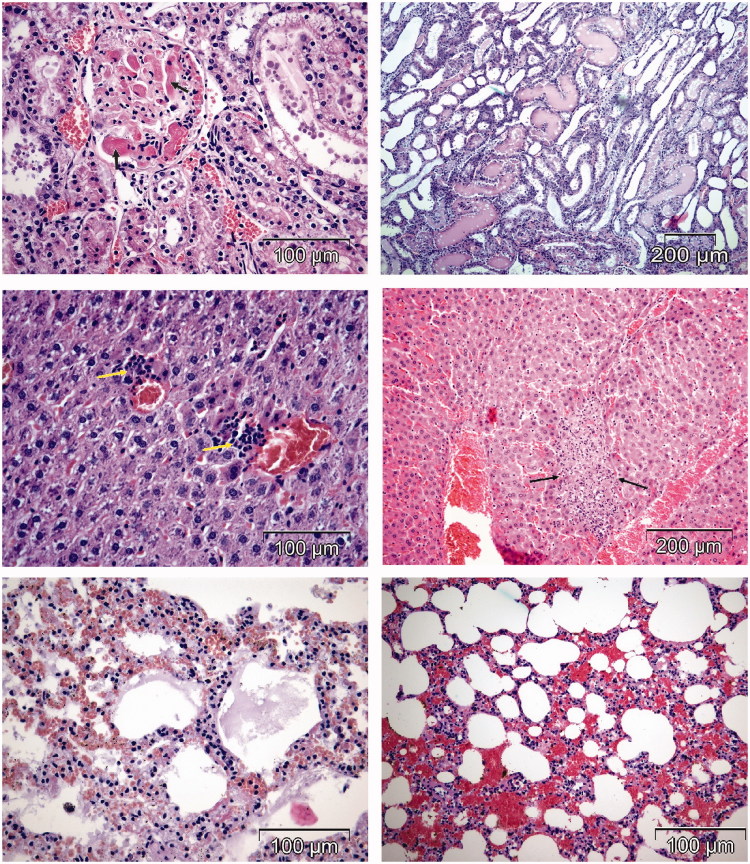
Organ toxicity induced by free DOX (HE stain). Upper micrographs show interstitial hemorrhage in the kidney (left) and fibrin amid deposits (right). Middle micrographs show areas of focal necrosis in the liver. Lower micrographs show pulmonary stasis (left) and hemorrhage (right).

**Figure 3.**
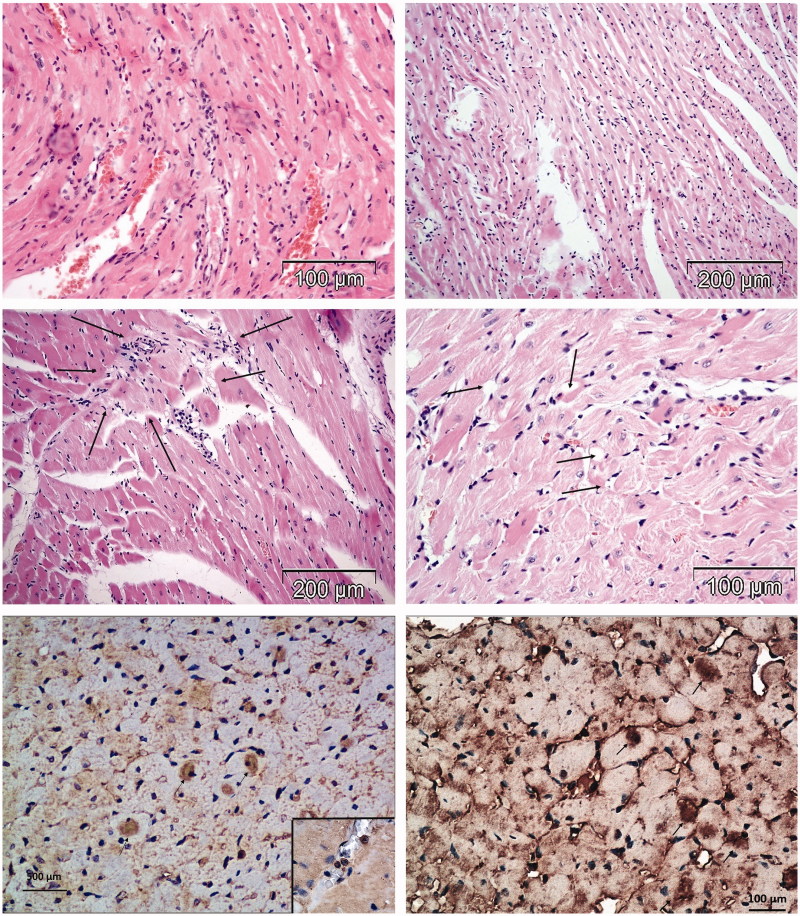
Histomorphological changes in the hearts of rats treated with DOX (HE stain). Rats treated with DOX had typical signs of cardiotoxicity: interstitial mononuclear infiltration, degeneration and diffuse necrosis of cardiomyocytes (upper left micrograph), loss of striation and interstitial hypercellularity (upper right micrograph); interstitial fibrosis (middle left micrograph) and vacuolar degeneration of cardiomyocytes (middle right micrograph). Lower micrographs illustrate expression of caspase 3 for detection of apoptosis in the heart tissue (insert shows lymphocytes).

All rats treated with free DOX were adynamic with ruffled fur, progressively losing weight and exhibiting cachexia with chromodacriorhea (Figure 1). Necropsy and histopathological examination revealed organ toxicity: glomerulosclerosis, tubulointerstitial inflammation and fibrosis in kidneys, focal necrosis in liver, congestion and hemorrhage in lungs (Figure 2), and loss of myofibrillar striation and vacuolar degeneration of cardyomyocites (Figure 3). Cytoplasmic immunostaining of cardiomyocytes to caspase 3 in DOX-treated rats revealed apoptosis (Figure 3, lower micrographs).

**Figure 4.**
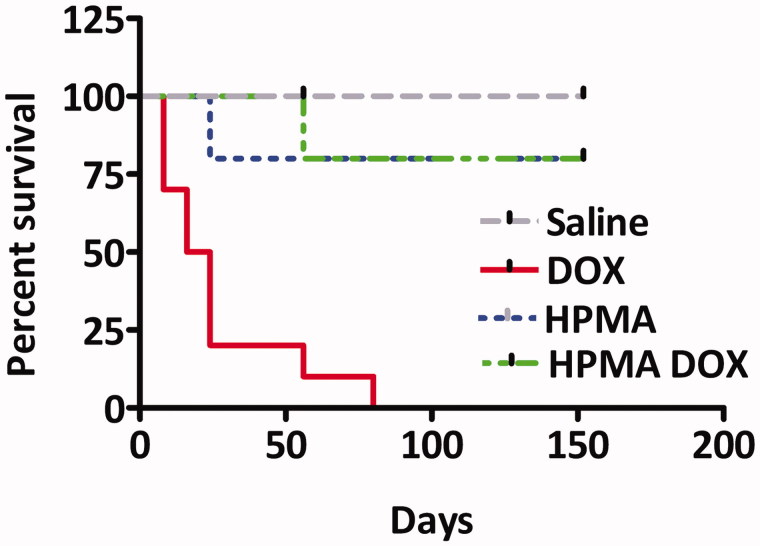
Survival of Wistar rats treated with different compounds. Note that only rats treated with free DOX exhibited low survival. Slightly and statistically insignificant lower survival of HPMA and HMPA–DOX rats in respect to SALINE treated rats was unrelated to treatment.

The survival rate of DOX-treated rats was low, and rats died between days 25 and 80 with 49 days median survival (Figure 4). The cause of death was either hepato-renal toxicity (*n* = 8) or cardiac toxicity (*n* = 3).

**Figure 5.**
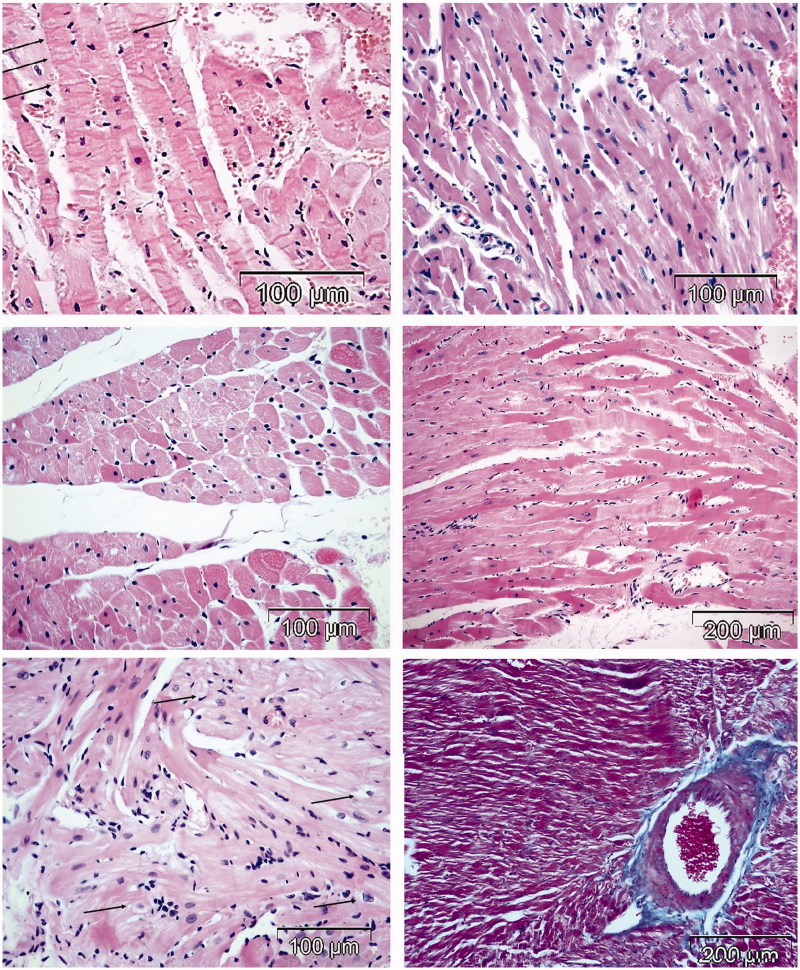
Histomorphological changes in the hearts of two rats treated with HPMA copolymer–DOX conjugate (HE and Masson’s Trichrome stain). Two rats treated with HPMA–DOX showed signs of cardiotoxicity: contraction band necrosis (upper left micrograph), diffuse interstitial infiltrate (lymphocytes and fibroblasts), focal necrosis (upper right micrograph), mild vacuolar degeneration of cardiomyocytes (left middle micrograph), loss of cross striation (middle right micrograph), vacuolar degeneration of cardyomyocites (lower micrograph on the left) and perivascular and interstitial fibrosis (right bottom micrograph Masson’s Trichrome staining).

**Figure 6.**
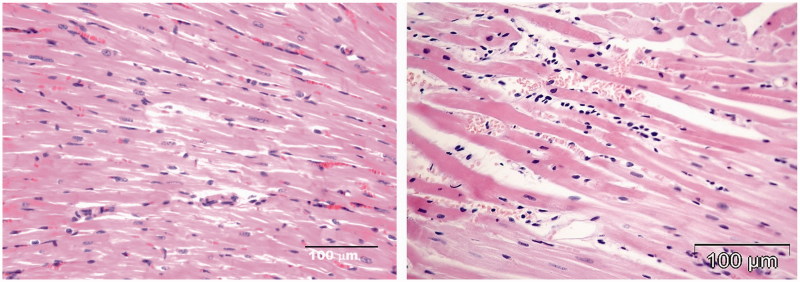
Histomorphological changes in the hearts of two rats treated with HPMA copolymer control (HE stain). Two rats treated with HPMA showed interstitial infiltration and hemorrhage (right micrograph). Left panel shows healthy myocardium in rats treated with saline.

Rats treated with HPMA copolymer–DOX, HPMA copolymer control and SALINE gained weight over time (Figure 1) and exhibited normal behavior. By the end of the follow-up period, only rats treated with HPMA copolymer–DOX exhibited unevenly flattened hair and areas of brownish fur coloration while SALINE and HPMA copolymer treated rats did not. Necropsy and histopathology revealed no organ toxicity in SALINE-treated rats. One rat treated with HPMA copolymer–DOX had signs of renal inflammation and hepatic venous dilation and two other rats exhibited cardiac toxicity (Figure 5). In two rats treated with HPMA copolymer alone, intra-cardiac hemorrhage and interstitial infiltration were observed (Figure 6). Also, one rat treated with HPMA copolymer and another with HPMA copolymer–DOX died before the end of the study due to peritoneal inflammation at the site of the implantation of radiotelemetric probe. Although these rats were excluded from the study, they were considered for the calculation of the mortality.

### Heart function and morphology by echocardiography in rats treated with different compounds

**Figure 7.**
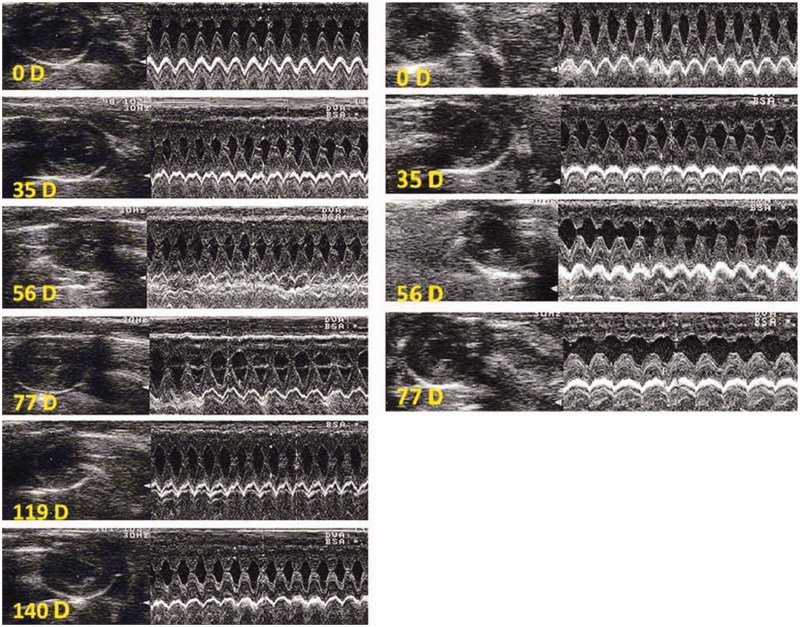
Echocardiography in one rat treated with DOX (right) and another with saline (left). B mode is shown at left and M mode at right. 0D indicates the day before treatment. Note that on day 56D and 77D, a rat treated with DOX exhibited increased left ventricular internal diameter in systole (LVIDs) and distole (LVIDd), enlarged posteror wall thickness (PW) and decreased interventricular septum thickness.

**Figure 8.**
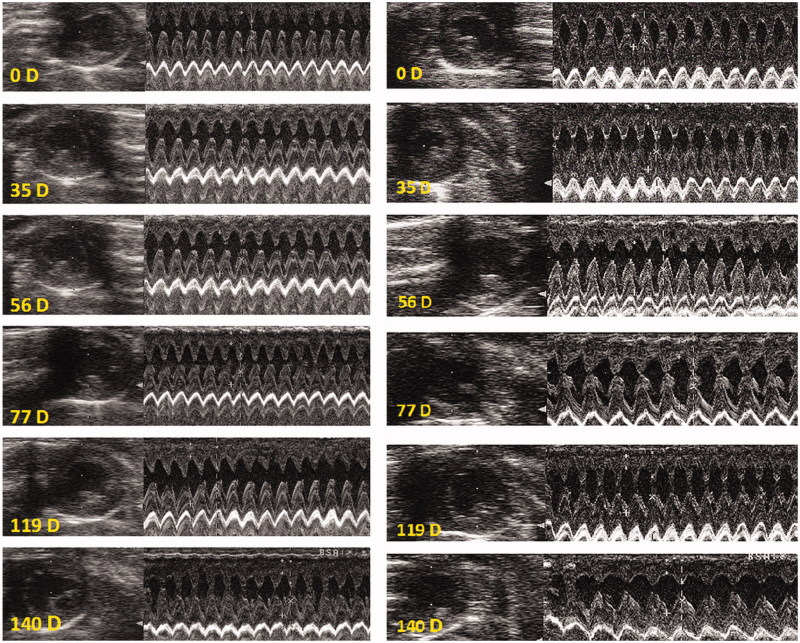
Echocardiography of one rat treated with HPMA copolymer (left) and another rat treated with HPMA copolymer–DOX conjugate (right). B mode is shown at left and M mode at right. Note an increase in left ventricular internal diameter in systole and diastole on day 140 (140D) in HPMA copolymer–DOX-treated rat (right bottom).

Three rats treated with DOX showed echocardiographic changes (Figure 7) while other rats died from non-cardiac causes before developing overt heart failure (Table 1) (refer to discussion). Rats treated with HPMA copolymer–DOX displayed an averagely 50% increase EDV on day 140 (*p* < 0.05 compare to saline control), while left ventricular ejection fraction (EF_LV_) remained normal (Table 1, Figure 8). Interestingly, rats treated with HPMA copolymer had shown a 25% increase EDV between 48 and 80 days after treatment, while EF_LV_ increased by 3% on day 140 after treatment (Table 1).

**Table 1.** Effects of different compounds on left ventricular end diastolic volume and ejection fraction.

	Day 0	Days 8–40	Days 48–80	Day 140
EDV (ml)				
SALINE	0.4 ± 0.02	0.4 ± 0.04	0.4 ± 0.05	0.4 ± 0.03
HPMA	0.4 ± 0.01	0.4 ± 0.03	0.5 ± 0.05*	0.5 ± 0.07*
HPMA–DOX	0.3 ± 0.04	0.4 ± 0.05	0.4 ± 0.06	0.6 ± 0.09**
DOX				
EF_LV_ (%)				
SALINE	88 ± 2	88 ± 2	88 ± 2	90 ± 3
HPMA	90 ± 1	89 ± 5	92 ± 1	93 ± 0.8*
HPMA–DOX	91 ± 0.8	91 ± 2	91 ± 9	88 ± 3
DOX	91 ± 1	91 ± 1	–	–

Values are mean of atleast six experiments ± SEM. EDV: left ventricular end diastolic volume; EF_LV_: left ventricular ejection fraction; SALINE: 0.9% NaCl; HPMA: *N*-(2-Hydroxypropyl) methylacrylamide copolymer; HPMA–DOX: doxorubicin conjugated to *N*-(2-Hydroxypropyl) methylacrylamide copolymer; DOX: doxorubicin. *p < 0.05; **p < 0.01 vs. Day 0 (before treatment).

### Heart histopathology in rats treated with different compounds

In all the rats treated with DOX (Figure 3) and two rats treated with HPMA copolymer–DOX (Figure 5), typical signs of DOX cardiotoxicity were observed: apoptosis, vacuolar degeneration of myocardiocytes, interstitial mononuclear infiltration of cardiac tissue, myofibrilar contraction band necrosis and interstitial hypercellularity and fibrosis. Two rats treated with HPMA copolymer control showed interstitial infiltration and hemorrhage (Figure 6).

### Hemodynamic and spectral changes in rats treated with different compounds

**Figure 9.**
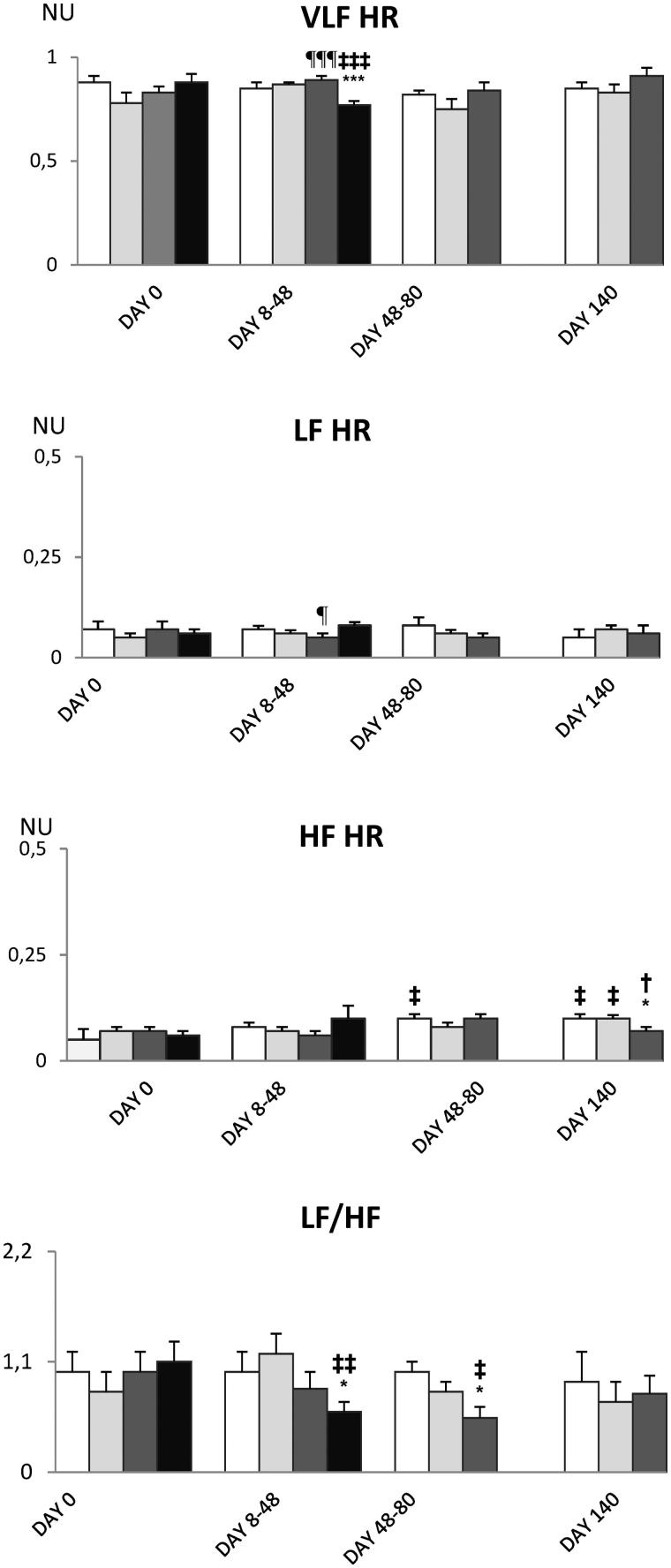
Effects of different compounds on the components of HR short-term varaibility. Empty bars indicate saline-treated rats, light gray bars and dark gray bars indicate HPMA- and HPMA–DOX-treated rats, respectively. Black bars represent rats treated with DOX. NU on Y-axis stands for normalized units. Note a decrease in VLF and LF/HF in DOX-treated rats. Experiments are mean of atleast six experiments ± SEM. ‡*p* < 0.05 vs. day 0; *p < 0.05 vs. saline; †*p* < 0.05 vs. HPMA; ¶*p* < 0.05 vs. DOX.

**Figure 10.**
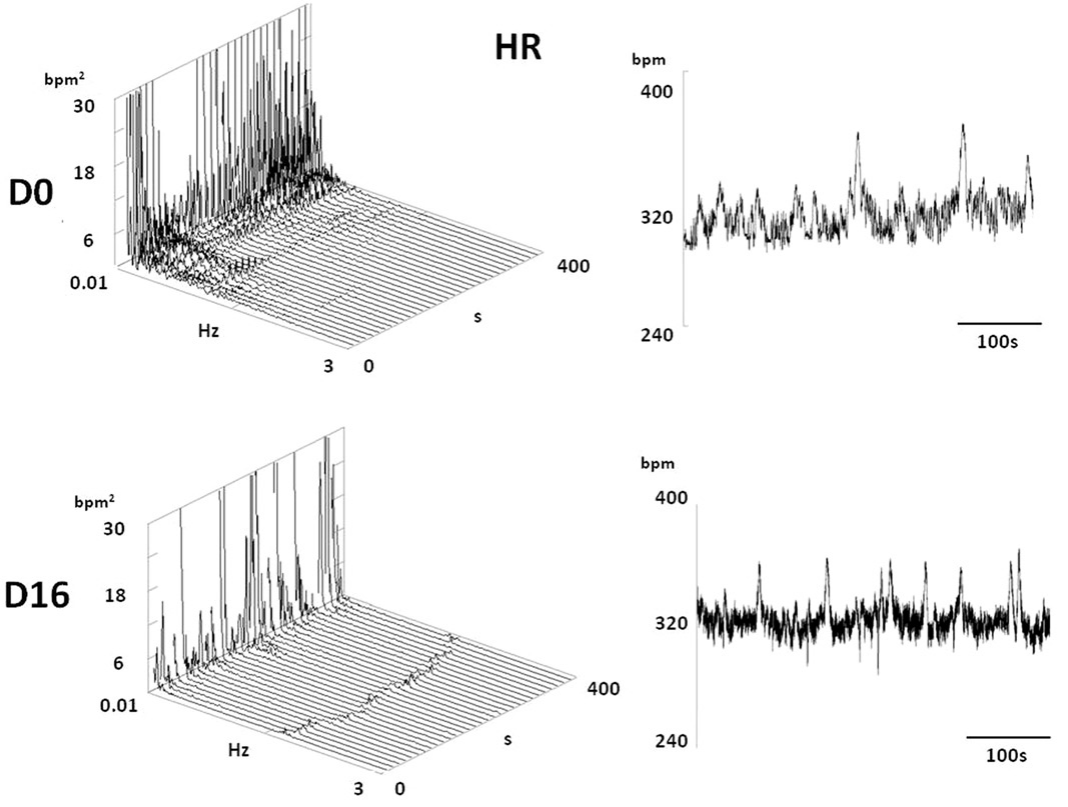
A typical HR spectrum of one rat before and after treatment with DOX. Note a decrease of spectral power in lower frequencies (VLF and LF) 16 days after treatment by DOX.

**Figure 11.**
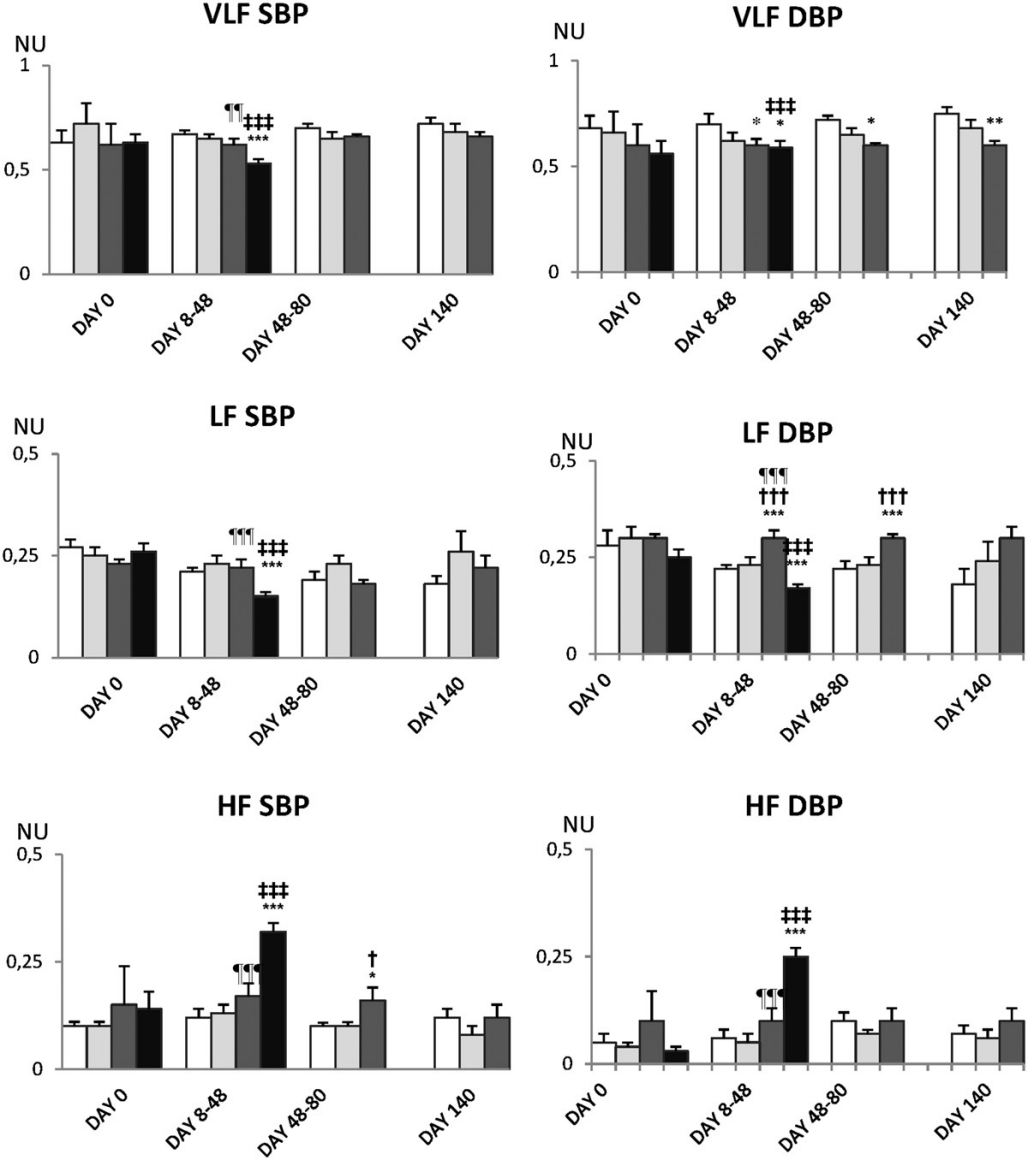
Effects of different compounds on the components of BP short-term variability. Empty bars indicate saline-treated rats, light gray bars and dark gray bars indicate HPMA- and HPMA–DOX-treated rats, respectively. Black bars represent rats treated with DOX. NU on Y-axis stands for normalized units. Note redistribution of spectral power towards HF in DOX-treated rats. Experiments are mean of atleast six experiments ± SEM. ‡*p* < 0.05 vs. day 0; *p < 0.05 vs. saline; †*p* < 0.05 vs. HPMA; ¶*p* < 0.05 vs. DOX.

**Figure 12.**
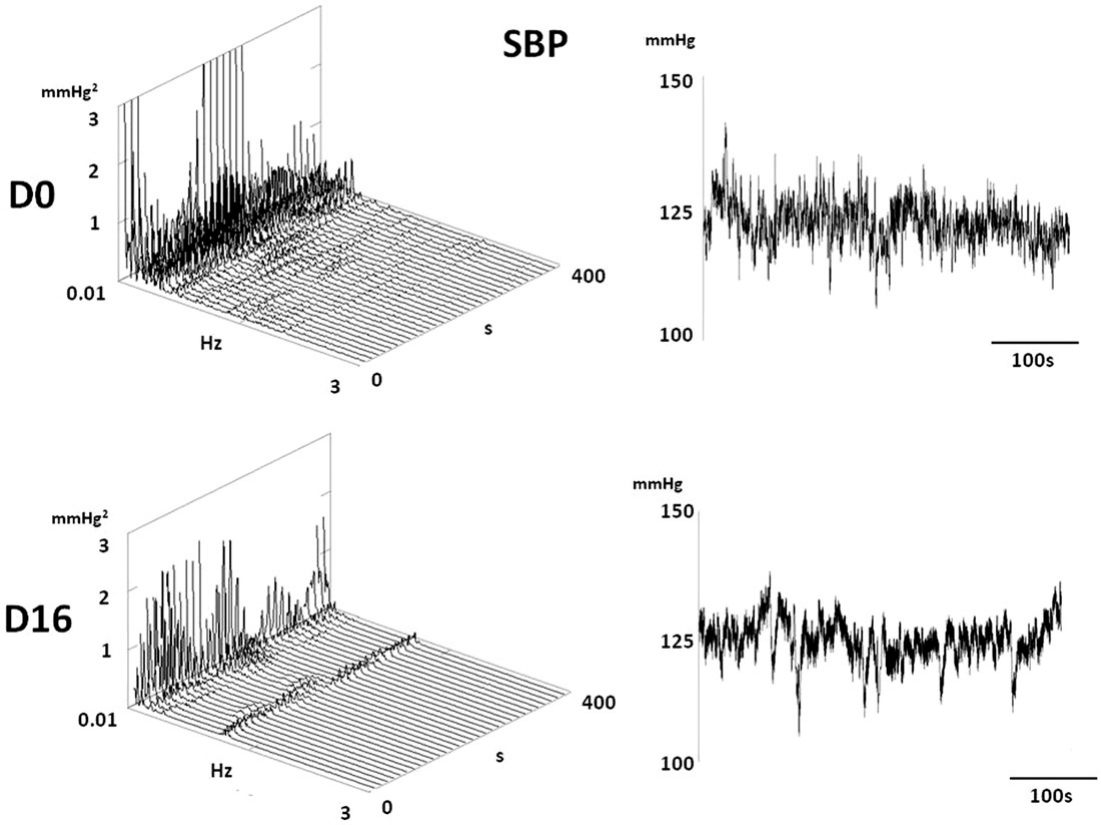
A typical SBP spectrum of one rat before and after treatment with DOX. Note a decrease of spectral power in lower frequencies (VLF and LF) in rats 16 days after treatment by DOX.

Table 2 shows that mean values of HR, SBP, MBP and DBP do not differ between experimental groups except for the slight increase in DBP in DOX-treated rats. Decreased HRV in VLF and LF domain as well as of the LF/HF ratio (Figures 9 and 10) predicted bad outcome in DOX-treated rats. BPV was also reduced in DOX-treated rats in VLF and LF domains and the spectral power of SBP was redistributed towards HF spectral range (Figures 11 and 12).

**Table 2.** Effects of different compounds on heart rate and arterial blood pressure.

	Day 0	Days 8–40	Days 48–80	Day 140
HR (bpm)				
SALINE	306 ± 8	313 ± 10	288 ± 8	287 ± 10
HPMA	344 ± 33	346 ± 32	330 ± 16	336 ± 16
HPMA–DOX	313 ± 17	314 ± 11	301 ± 9	307 ± 10
DOX	328 ± 13	327 ± 15	–	–
MBP (mmHg)				
SALINE	94 ± 3	97 ± 5	94 ± 3	92 ± 4
HPMA	94 ± 4	93 ± 6	92 ± 7	94 ± 6
HPMA–DOX	91 ± 4	93 ± 5	96 ± 6	95 ± 9
DOX	96 ± 4	104 ± 4	–	–
SBP (mmHg)				
SALINE	117 ± 3	121 ± 2	119 ± 3	117 ± 3
HPMA	117 ± 5	119 ± 8	115 ± 8	120 ± 9
HPMA–DOX	112 ± 4	116 ± 5	119 ± 6	120 ± 8
DOX	119 ± 2	120 ± 4	–	–
DBP (mmHg)				
SALINE	83 ± 2	85 ± 3	82 ± 2	79 ± 4
HPMA	82 ± 3	80 ± 4	80 ± 6	81 ± 3
HPMA–DOX	81 ± 4	81 ± 5	84 ± 6	83 ± 10
DOX	85 ± 2	96 ± 5‡*	–	–

Values are mean of atleast six experiments ± SEM. MBP: mean blood pressure; SBP: systolic blood pressure; DBP: diastolic blood pressure. SALINE: 0.9% NaCl; HPMA: *N*-(2-Hydroxypropyl) methylacrylamide copolymer; HPMA–DOX: doxorubicin conjugated to *N*-(2-Hydroxypropyl) methylacrylamide copolymer; DOX: doxorubicin. *p < 0.05 vs. SALINE; ‡*p* < 0.05 vs. Day 0 (before treatment).

## Discussion

This is the first comprehensive hemodynamic cardiovascular study in conscious rats that shows improved cardiovascular tolerability of HPMA copolymer–DOX conjugate in respect to the free drug. HPMA copolymer–DOX conjugate treated rats had better survival, lower organ toxicity and exhibited no changes in BPV and HRV. In contrast, DOX-treated rats exhibited reduction in BPV and HRV which predicted *exitus letalis*. In few, not all rats treated with HPMA copolymer alone, echocardiography finding and histopathology examination noted cardiac injury, recommending caution with the use of HPMA copolymer drug carrier system.

Observation of the general condition of rats treated with free DOX revealed they were progressively losing weight until developing cachexia, that they were adynamic, and that the survival rate was significantly lower in respect to rats treated with HPMA copolymer–DOX conjugate. HPMA copolymer–DOX conjugate-, HPMA copolymer control- and SALINE-treated rats gained weight over time and survived throughout the follow-up period suggesting improved tolerability upon DOX conjugation. The cause of death in rats treated with free DOX was due to multiple organ toxicity. Necropsy revealed toxic nephropathy and focal liver necrosis in all rats treated with free DOX. As expected, free DOX induced cardiomyopathy, and associated changes in lungs and liver such as pulmonary and hepatic stasis. The heart tissue of all rats treated with free DOX showed typical signs of cardiotoxicity: apoptosis and vacuolization of cardiomyocytes, inflammation, focal necrosis and loss of cross striation. However, only two rats treated with polymer bound drug exhibited cardiotoxic changes, and, along with vacuolar degeneration of cardiomyocytes, loss of cross striation, focal necrosis and inflammation, they also showed signs of reparation such as perivascular fibrosis. HPMA copolymer–DOX conjugate-treated rats had no toxic changes in liver, and only one rat had affected kidneys. Present findings confirm previous reports on DOX-induced general toxicity (Bertinchant et al., 2003; Lee & Harris, 2011; Mitry & Edwards, 2016; Octavia et al., 2012) and also confirm improved cardiac tolerability of HPMA copolymer–DOX conjugate in respect to free DOX (Duncan et al., 1998; Hopewell et al., 2001; Yeung et al., 1991). The plausible explanation for better tolerability of HPMA copolymer–DOX conjugate in respect to the free drug is possibly due to the high molecular weight of the drug carrier HPMA copolymer and lower permeability of end-organs’ vasculature to macromolecules (Kedar et al., 2010; Kopeček et al., 2000). It is important to note that HPMA copolymer–DOX conjugate has a longer half-life than free DOX (Tomalova et al., 2016) and together with time needed for the release of the drug from the drug carrier to become active, it is reasonable to expect that pharmacological and toxicological effects may be modified and delayed. That is why the follow up period in this study was double the time of survival of rats treated with free DOX. Additionally, DOX conjugated to HPMA is not pharmacologically active (Malugin et al., 2007), and, unlike free DOX that enters cells by diffusion, HPMA copolymer–DOX conjugate is up-taken into cells by endocytosis. Only when endocytotic vesicle fuses with lysosomes, DOX is enzymatically released in the cell by cleavage of peptide spacers as cathepsin B substrates, and then exerts pharmacological effects (Duncan & Vicent, 2010; Kedar et al., 2010). Subsequently, HPMA copolymer is trafficked to late endosomes and lysosomes for degradation. In most of the circumstances, many of them may accumulate in vesicle within the cell instead of being degraded by the lysosomal environment (Fox et al., 2009). This accumulation could result in toxicity over the time. Our study revealed that two rats treated with HPMA copolymer alone, showed interstitial infiltration and hemorrhage in the heart tissue, suggesting the possibility of HPMA copolymer accumulation may lead to heart damage. Altogether the present observations suggest improved tolerability of HPMA copolymer–DOX conjugate in respect to free drug but also prudence with the use of HPMA copolymer for drug delivery.

In this study, identification of the onset of heart failure and its progression was assessed non-invasively using echocardiography because it could be repeated as often as needed and because it has been approved for preclinical drug safety and toxicological assessment (Hanton et al., 2008). EDV and left ventricular EF_LV_ were taken as markers of overt heart failure. Only three rats, out of eleven rats treated with DOX, developed overt heart failure (Figure 4). Other rats died from other causes before developing signs of heart failure. In another study in rats treated with free DOX, a greater incidence of heart failure was reported using echocardiography (Teraoka et al., 2000). The discrepancy with the present study could be explained by the fact that Teraoka and collaborators (2000) carried out echocardiography in anesthetized rat while we performed echocardiography in conscious sedated rats. The inhibitory effect of anesthesia on autonomic cardiac performance is well established as well as the potentiating effect of anesthesia on negative inotropism (Stein et al., 2007). Acepromzine used to sedate the rats in the present study, is a phenothiazine that does not exert negative inotropic effects, but reduces peripheral resistance due to α_1_ blocking properties (Algren & Ashworth, 2015) improve heart performance. However, concomitant administration with DOX is not recommended in veterinary practice (not the case here) to avoid excessive perivascular leak of DOX and DOX-induced vascular injury and sclerosis. Another intriguing finding is that, by the end of follow-up period, rats treated with HPMA copolymer–DOX conjugate exhibited about 50% increase in EDV without changes in left ventricular EF_LV_. This finding could be an early manifestation of postponed heart failure, as EDV is a more sensitive indicator of heart failure than left ventricular EF_LV_. Additionally, EDV increased by about 25% in rats treated with HPMA copolymer alone and this was accompanied by small (3%) but statistically significant (Table 1) increase of left ventricular EF_LV_. The increase in EDV and EF_LV_ could be attributed to HPMA’s plasma expander properties (Kopeček et al., 1973) and the increase in circulating volume and heart filling. Alternatively, EDV increase could be induced by the weakening and dilatation of the left ventricular wall due to intra-mural hemorrhage.

HPMA copolymer–DOX conjugate and DOX did not affect mean values of mean BP and HR. However, in DOX-treated rats spectral analysis of HR indicates a decrease in HRV in lower spectral frequencies and a decrease of the LF to HF HR ratio. The decrease in HRV has been shown to be a bad prognostic sign in heart failure patients (Ponikowski et al., 1997) but also in patients suffering from hepatic failure (Haddadian et al., 2013; Mani et al., 2009) and renal failure (Drawz et al., 2013), all of which have been detected in DOX-treated rats. HR variability is created by the activity of the autonomic nervous system and to a much lesser extent by the intrinsic heart mechanisms (Japundzic et al., 1990). Under baseline physiological conditions, HRV is under dominant vagal control depicted in the high frequency (HF) spectral range. During stressful challenges, sympathetic nervous system impinges on HR variability and increases low frequency (LF) spectral band (Šarenac et al., 2011; Stojičić et al., 2008). Thus, LF/HF HR ratio increase indicates a shift of sympatho-vagal balance in favor of the sympathicus during stress. Conversely, a decrease in HR variability denotes impaired autonomic control of the heart (Ponikowski et al., 1997) and this was found to occur in autonomic neuropathy accompanying diverse diseases (Ranpuria et al., 2008; Stuckey & Petrella, 2013). In present experiments, it is unlikely that a decrease in HRV was induced by DOX-induced autonomic neuropathy as it is uncommon with DOX (Miltenburg & Boogerd, 2014). The possibility is that down-regulation of adrenergic β-receptors reported to occur in the hearts of rats treated with DOX (Tong et al., 1991) reduced the responsiveness of the heart to normal sympathetic stimulation. Hence, reduction of HRV in lower frequency range and reduction of the LF/HF ratio in present experiments probably denotes reduced sensitivity of the heart in DOX treated rats to sympathetic nervous system stimulation.

In present experiments, DOX treated rats, but not HPMA copolymer DOX conjugate treated rats, exhibited a decrease in BPV in VLF and LF frequency ranges and redistribution of spectral power towards respiration-induced, HF range. The changes of BP variability were observed in rats exhibiting hepato-renal toxicity as well as in rats exhibiting heart failure. The spectral power of BP low frequencies is set by an interplay of the sympathetic nervous system, renin-angiotensin system (RAS) and locally produced vasoactive molecules such as NO, bradykinin etc. (Japundzic-Zigon, 1998). In present experiments reduction of VLF and LF BP variability denotes reduction in peripheral resistance. This could be either due to reduce sympathetic tone and RAS activity or increased production of vasodilator molecules. For instance, in hepatic failure increased production of nitric oxide, carbon monoxide, endogenous cannabinoids and other molecules that decrease vascular reactivity to vasoconstrictors, especially in splanchnic vascular bed, is well documented (Fede et al., 2014). Also increased expression of angiotensin converting enzyme type 2 has been reported to occurs in heart failure, as well as in hepatic and renal failures (Cohen-Segev et al., 2014; Patel et al., 2016). Increased level of ACE 2 and increased production of angiotensin 1–7 activates Mas receptors in arterial blood vessels that causes vascular hypocontractility and vasodilatation (Dharmani et al., 2007; Grace et al., 2013) that could contribute to the decrease of lower frequencies in BP spectra. There is also a possibility that DOX – induced damage of vascular endothelium contributed to changes in BPV (López-Miranda et al., 2010).

In present experiments, changes in BPV and HRV induced by DOX were associated to multiple organ damage and predicted bad outcome. These changes were not observed in HPMA copolymer–DOX conjugate-treated rats who exhibited lower organ toxicity and better survival. These findings support the use of spectral analysis of BPV and HRV as tools for preclinical risk assessment.

## Conclusions

Altogether present findings show that HPMA copolymer–DOX conjugate-treated rats do not affect cardiovascular short-term variability which predicted improved overall and cardiovascular tolerability in respect to free DOX-treated rats who exhibited reduced cardiovascular variability and low survival. HPMA copolymer–DOX conjugate-treated rats and rats treated with HPMA copolymer alone had increased EDV and some of them showed microscopic injury of the heart tissue. Therefore, prudence is suggested with the use of HPMA copolymer system for drug delivery. Finally, our observations validate the use of echocardiography and radio-telemetry along with spectral analysis of cardiovascular short-term variability for preclinical risk assessment of polymer drug conjugates in toxicology and safety pharmacology.

## Supplementary Material

Supplementary Material
