# Supplementary figures and images for: Hemodynamic effects of HPMA copolymer based doxorubicin conjugate: A randomized controlled and comparative spectral study in conscious rats
Source: Nanotoxicology. 2017 Feb 9;11(2):210–22. doi: 10.1080/17435390.2017.1285071 (PMC5964453; doi:10.1080/17435390.2017.1285071)

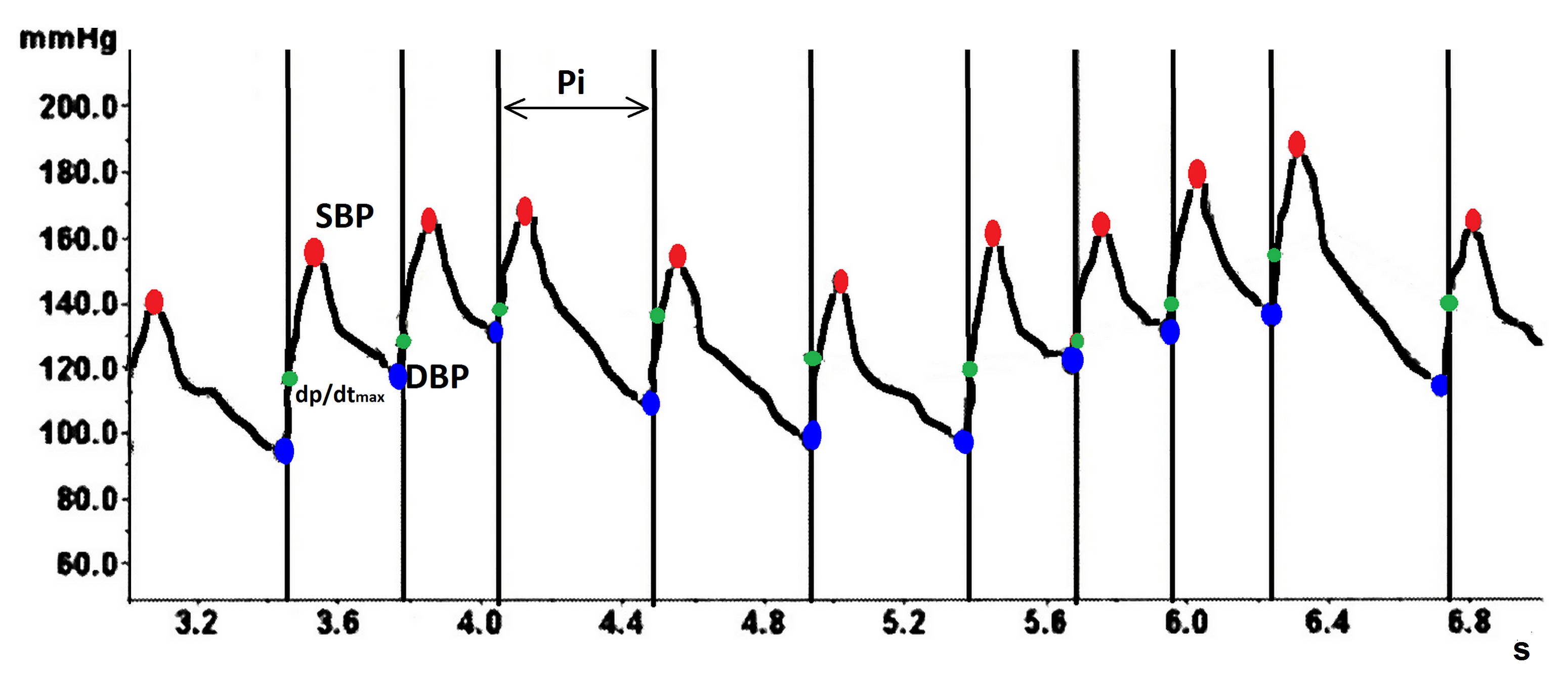

Supplement: Supplementary Material [file INAN_A_1285071_SM2814.tif]
